# Bibliographical

**Published:** 1856-06

**Authors:** 


### BIBLIOGRAPHICAL.

We announced in our last number the reception of a num-of medical works from Messrs. Blanchard & Lea, of Philadelphia, through the hands of Messrs. J. J. Richards & Co., of this city. In the first place, we have "Flint on the Respiratory Organs, the Physical Exploration and diagnosis of their diseases," the object of which seems to be to supply a "desideratum, viz., a work limited to diseases effecting the Respiratory Organs, treating *in extenso*, and almost exclusively, of the principles and practice of physical exploration, as applied to the diagnosis of these affections."

The intention of the author seems to have been to make a practical work, and he has therefore considered the various topics with almost exclusive reference to their direct practical bearings. The author remarks, "Of most of the practical points embraced in this work, I am able to speak from experience; with respect to certain signs, the views which I have been led to form from personal observation, *are original.*" He then particularizes characters of pitch distinguishing the respiratory sound commonly called *rude*, or rough, and which give to a prolonged expiration its significance as a sign of increased density of lung from tubuculous or other solid deposit; also the relative pitch of the inspiratory and expiratory sounds in the cavernous, as contrasted with the bronchial, respiration, other points mentioned, are the importance of determining the line of the interlobulæ fissure, as a means of distinguishing between the percussion dullness of lobar-pneumonitis and liquid effusion, and the clinical value of the *souffle* or bellows sound, accompanying the act of whispering, as a sign of solidification. Whilst deprecating needless distinctions and overrefiements in principles and practice, and mutations in classification and nomenclature, he has nevertheless "ventured to propose the substitution of a new name for *rude* or *rough* respiration, viz., *broncho-vesicular*, and the use of the terms *vesiculo-tympanitic* resonance, applied to a percussion-sound combining the tympanitic and vesicular qualities, and *broncho-cavernous* respiration, denoting a mixture of the cavernous and bronchial modifications of the respiratory sound.

The work contains 628 pages, embracing an introduction with two sections devoted to "Preliminary points pertaining to the Anatomy and Physiology of the Respiratory Apparatus," and "Topographical Divisions of the Chest," with two Parts; the first devoted to "Physical Exploration of the Chest," comprising "Definitions," "Different methods of Exploration," "Percussion," "Auscultation," "Inspection," "Mensuration," "Palpation," "Succussion," "Recapitulatory enumeration of the Physical signs furnished by the several methods of Exploration," and "Correlation of Physical Signs," making up a little more than half the work. In the second part he presents the "Diagnosis of Diseases affecting the Respiratory Organs," including chapters on "Bronchitis, Pulmonary or Bronchial Catarrh," "Dilatation and Contraction of the Bronchial Tubes—Pertussis Asthma," "Pneumonitis—Imperfect Expansion (Atelectasis) and Collapse," "Emphysema," "Pulmonary Tuberculosis—Bronchial Pthisis," "Pulmonary Œdema Gangrene of the Lungs—Pulmonary Apoplexy—Cancer of the Lungs—Cancer in the Mediastinum," "Acute Pleuritis—Chronic Pleuritis—Empyema—Hydrothorax—Pneumothorax—Pneumo-Hydrothorax—Pleuralgia—Diaphragmatic Hernia," "Diseases affecting the Larynx and Trachea Foreign bodies in the Air Passages," and, in addition, is attached a short "Appendix" "On the Pitch of the Whispering Souffle over Pulmonary Excavations;" and upon the whole we have higher authority than our own, for saying that the "character of the work is such, that it cannot fail to raise him high in the opinion of his medical brethren, as an exact and conscientious observer." The author is Professor Austin Flint of the University of Louisville.

We have also received a work of 276 pages, "On some of the Diseases of Women admitting of surgical treatment, by

Isaac Baker Brown, F. R. C. S., (By Exam.) Surgeon Accoucheur to St. Mary's Hospital, Vice President of the Medical Society of London, &c., &c.," from the same publishers, through the same hands.

As the author remarks, "There is no branch of surgery more open to improvement than that which relates to those accidents and diseases incident to the female sex, which admit of no relief except from the hand of the Surgeon. In the standard works on Midwifery and the Diseases of Females, these surgical diseases are for the most part but imperfectly discussed, and their treatment is often described in a few words, and without any suggestions to direct the surgeon through the difficulties and dangers of the more important operations proposed for their relief." It is to the diagnosis and treatment of those cases, about which little information is found in the books, that our author has devoted the bulk of his volume, though adding some details of practical importance to those that have been elaborately treated elsewhere.

The book is divided into two sections; the first embracing Diseases or accidents which result directly or indirectly from Parturition, and under the second section are classed, Diseases or accidents of the female organs occurring independently of Pregnancy. Under the first section are placed *Operations* for Rupture of the Perineum, Prolapsus Vaginæ, Prolapsus and Procidentia Uteri, Verico Vaginal Fistula, Recto Vaginal Fistula, and Lacerated Vagina. Under the second section are placed *Operations* for Polypus Uteri, Stone in the Female Bladder, Vascular Tumor of the Meatus Urinarius, Imperforate Hymen, Encysted Tumors of the Labia, Diseases of the Rectum resulting from certain conditions of the Uterus, and Ovarian Tumors. We are sorry to say that it has as yet been impossible for us to read the work, as from a slight glance we are satisfied that it is an exceedingly valuable contribution to surgical science.

Our thanks are also due to Messrs. Blanchard & Lea for a most admirable "Atlas of Cutaneous Diseases, by J. Moore, Neligan, M. D., Edin. M. R. I. A., &c., &c., which we have no hesitation in saying should belong to every student and practitioner of medicine, being a book of Plates of Cutaneous Eruptions, most beautifully, and, so far as we are capable of judging, most faithfully and accurately executed. We *know* that there is no class of diseases, in reference to which the junior practitioner has so much difficulty in making up a diagnosis. This work will be found an invaluable aid in enabling him to determine promptly and correctly the true character of Skin diseases; and thus contributing in the first degree to *their* successful treatment, as well as largely aiding in *his* professional advancement. In reference to "Neill & Smith's Compend of the various branches of Medical Science," it is useless for us to say any thing (it having already fully established itself in the warmest affections of students of medicine, for whom it has been mainly prepared,) farther than to add, that this is a new edition for March, 1856, with considerable modifications throughout all the departments, required by the numerous discoveries and improvements that have been made since the appearance of the previous edition.

Our individual thanks are also due to the same publishers for a copy of a "Manual of Chemical Physiology," from the German of Prof. C. G. Lehman, M. D., translated with notes and additions by Cheston Morris, M. D., with an Introductory Essay on Vital Force, by Samuel Jackson, M. D., Professor of Institutes of Medicine in the University of Pennsylvania, illustrated with forty wood cuts. To those who wish to ascertain what is established in Physiological Chemistry, without laborious investigation, probably no better book is offered; indeed, we believe that Lehman is considered the head and front of authority upon Modern Chemical Physiology. It is a work of *Science* proper, from the highest and most reliable source, and it is unnecessary to say more in the way of commendation.

We have also to acknowledge the receipt of "Curling on Diseases of the Testis," which will be noticed in a future number.

To Messrs. S. S. & W. Wood, No. 389 Broadway, New York, we are indebted for the April number of "The British and Foreign Medico-Chirurgical Review or Quarterly Journal of Practical Medicine and Surgery," published at $3 per annum—to aid in the circulation of which, we should consider as doing valuable service to the profession.

